# Ring-Over-Ring Deslipping From Imine-Bridged Heterorotaxanes

**DOI:** 10.3389/fchem.2022.885939

**Published:** 2022-05-03

**Authors:** Sayaka Hoshino, Kosuke Ono, Hidetoshi Kawai

**Affiliations:** ^1^ Department of Chemistry, Faculty of Science, Tokyo University of Science, Tokyo, Japan; ^2^ Department of Chemistry, Tokyo Institute of Technology, Tokyo, Japan

**Keywords:** dynamic covalent bond, imine, macrocycle, molecular shuttle, rotaxane, supramolecular chemistry

## Abstract

Ring-over-ring slippage and ring-through-ring penetration are important processes in the construction of ring-in-ring multiple interlocked architectures. We have successfully observed “ring-over-ring deslipping” on the rotaxane axle by exploiting the dynamic covalent nature of imine bonds in imine-bridged heterorotaxanes **R1** and **R2** with two macrocycles of different ring sizes on the axle. When the imine bridges of **R1** were cleaved, a hydrolyzed hetero[4]rotaxane **[4]R1′** was formed as an intermediate under dynamic equilibrium, and the larger 38-membered macrocycle **M** was deslipped over the 24-membered ring (24C8 or DB24C8) to dissociate into a [3]rotaxane **[3]R3** and a macrocycle **M**. The time dependent NMR measurement and the determined thermodynamic parameters revealed that the rate-limiting step of the deslipping process was attributed to steric hindrance between two rings and reduced mobility of **M** due to proximity to the crown ether, which was bound to the anilinium on the axle molecule.

## Introduction

**FIGURE 1:**
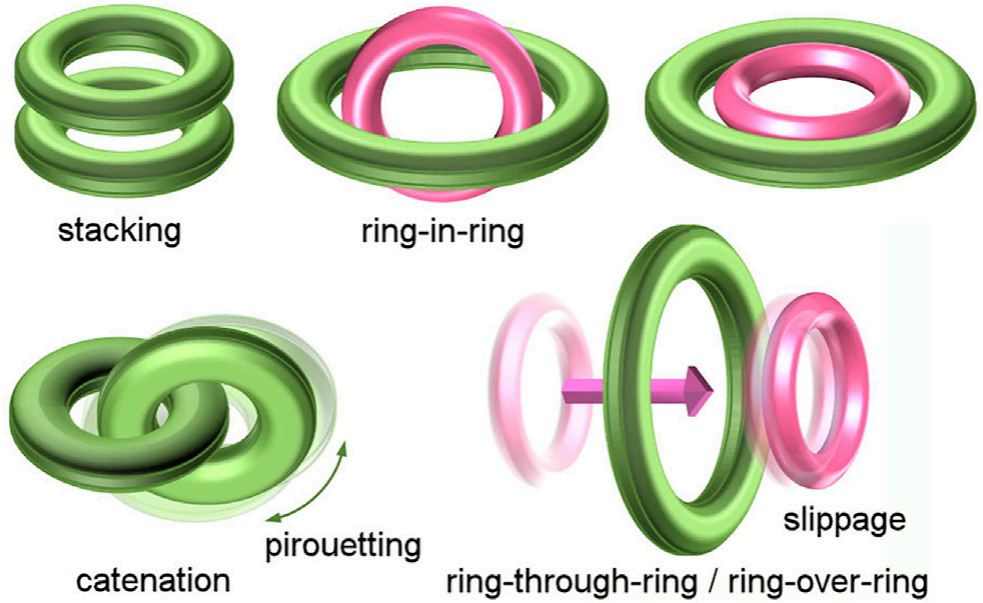
Interaction and mobility between ring and ring.

Cyclic molecules are chemical species that have attracted the interest of chemists due to their topology, restricted flexibility, and internal cavity (Forgan et al., 2011). The interaction and relative mobility, such as threading, slipping, and shuttling motion between ring and linear molecules, have been extensively studied by rotaxane chemistry (Amabilino and Stoddart, 1995; Kay et al., 2007; Xue et al., 2015). On the other hand, the interactions and motions between cyclic molecules have been investigated with regard to stacking (Grave and Schlüter, 2002) and “ring-in-ring” assembly (Cantrill et al., 2005; Kawase et al., 2007; Klosterman et al., 2016), catenation and pirouetting motion (Evans and Beer, 2014), while “ring-over-ring” slippage has been rarely investigated (Schweez et al., 2016; Zhu et al., 2018) (Figure 1). For example, the rings of main-chain [n]rotaxanes, which have multiple rings on the axle (Harada et al., 2009; Fang et al., 2010), usually do not slip past each other, but translate together. This limitation in mobility is the cause of sequence isomers in hetero[n]rotaxanes (Fuller et al., 2010; Neal and Goldup, 2014; Wang et al., 2018) and has been applied to the development of new rotaxane construction methods, such as cascade stoppering based on integrative threading of rings (Jiang et al., 2008; Rao et al., 2017). On the other hand, the first “ring-through-ring” rotaxane was reported by Loeb, where it was revealed that a [24]crown-8 ring (24C8) could pass through a [42]crown-8 ring but not a [30]crown-8 one on the axle (Zhu et al., 2018). This “ring-through-ring” slipping has opened a new gate to extend the mobility range between components inherent in rotaxane.

**FIGURE 2:**
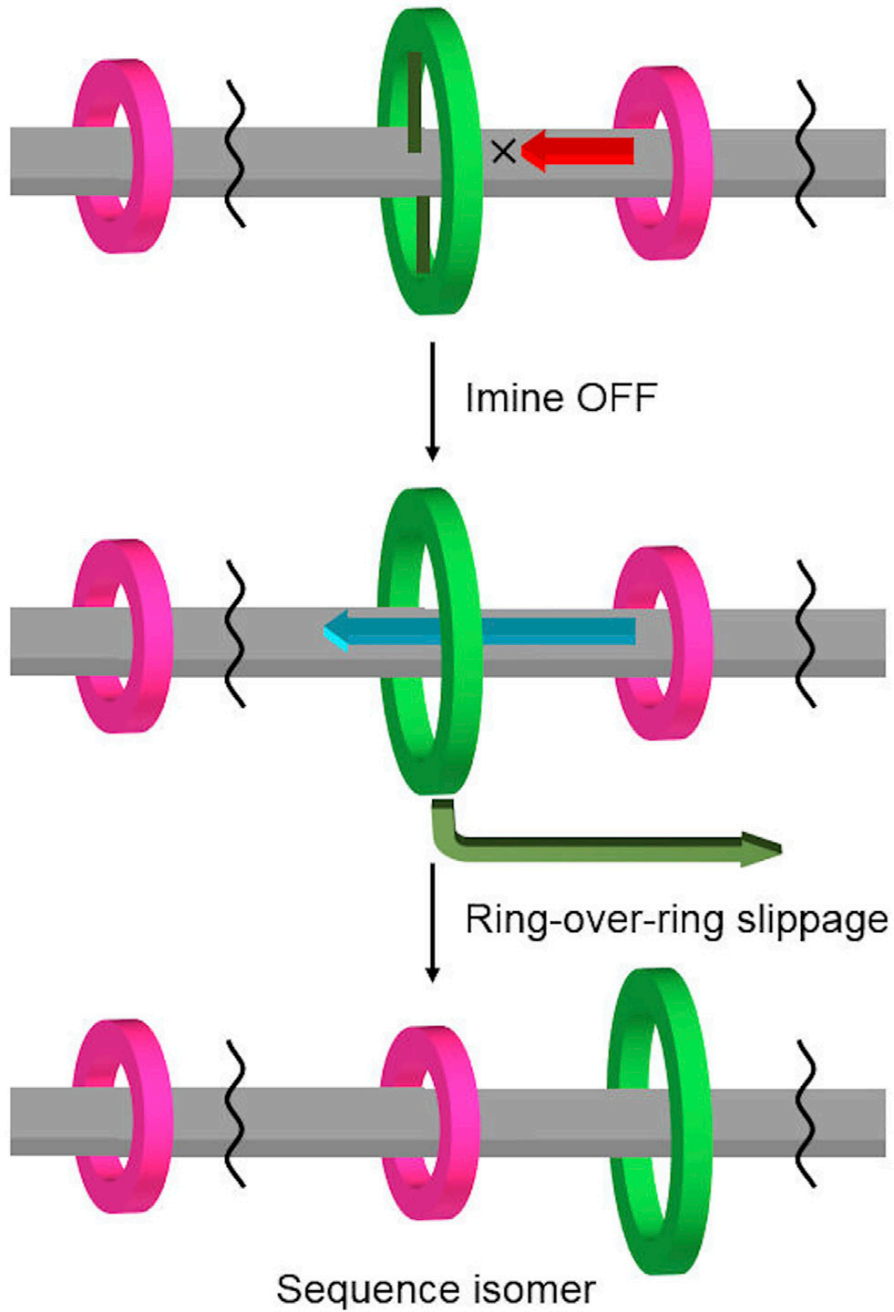
Gating control of ring position and mobility in hetero[n]rotaxane using a combination of imine-bridging and “ring-over-ring” slippage.

We have developed imine-bridged rotaxanes in which the mobility of the rings can be switched reversibly by turning on/off the imine bridges between the aniline ring and the axle of the imine-bridged rotaxane (Kawai et al., 2006; Umehara et al., 2008; Sugino et al., 2012). We predicted that if additional smaller rings were placed on the axle of this imine-bridged rotaxane, the dynamic covalent bond between the central aniline macrocycle and the axle would act as a gate for the shuttling motion of the smaller rings, allowing gating control (Chatterjee et al., 2006; Erbas-Cakmak et al., 2015; Borsley et al., 2021) of the position and mobility of rings (Figure 2). To use this control method, the following requirements must be achieved: 1) the incorporation of an additional small ring into the imine-bridged rotaxane by using different interactions; 2) the ability of the larger aniline macrocycle to pass over the small ring (or the small ring to pass through the larger macrocycle); and 3) the stability and reversibility of the imine bridging site in the heterorotaxane for keeping the aniline ring connected to the axle and releasing its mobility. As a part of our efforts toward this target, we have successfully observed “ring-over-ring deslipping” from the rotaxane axle, where the large macrocycle surmounts the small ring, by controlling the imine bridging of a hetero[4]rotaxane with two types of macrocycles with different ring sizes.

**SCHEME 1:**
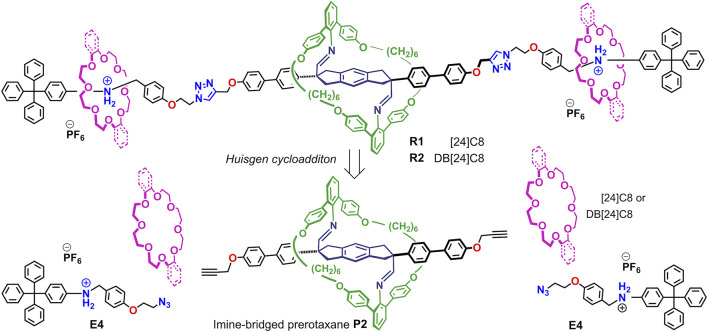
Synthetic strategy of imine-bridged heterorotaxanes **R1** and **R2**.

**SCHEME 2:**
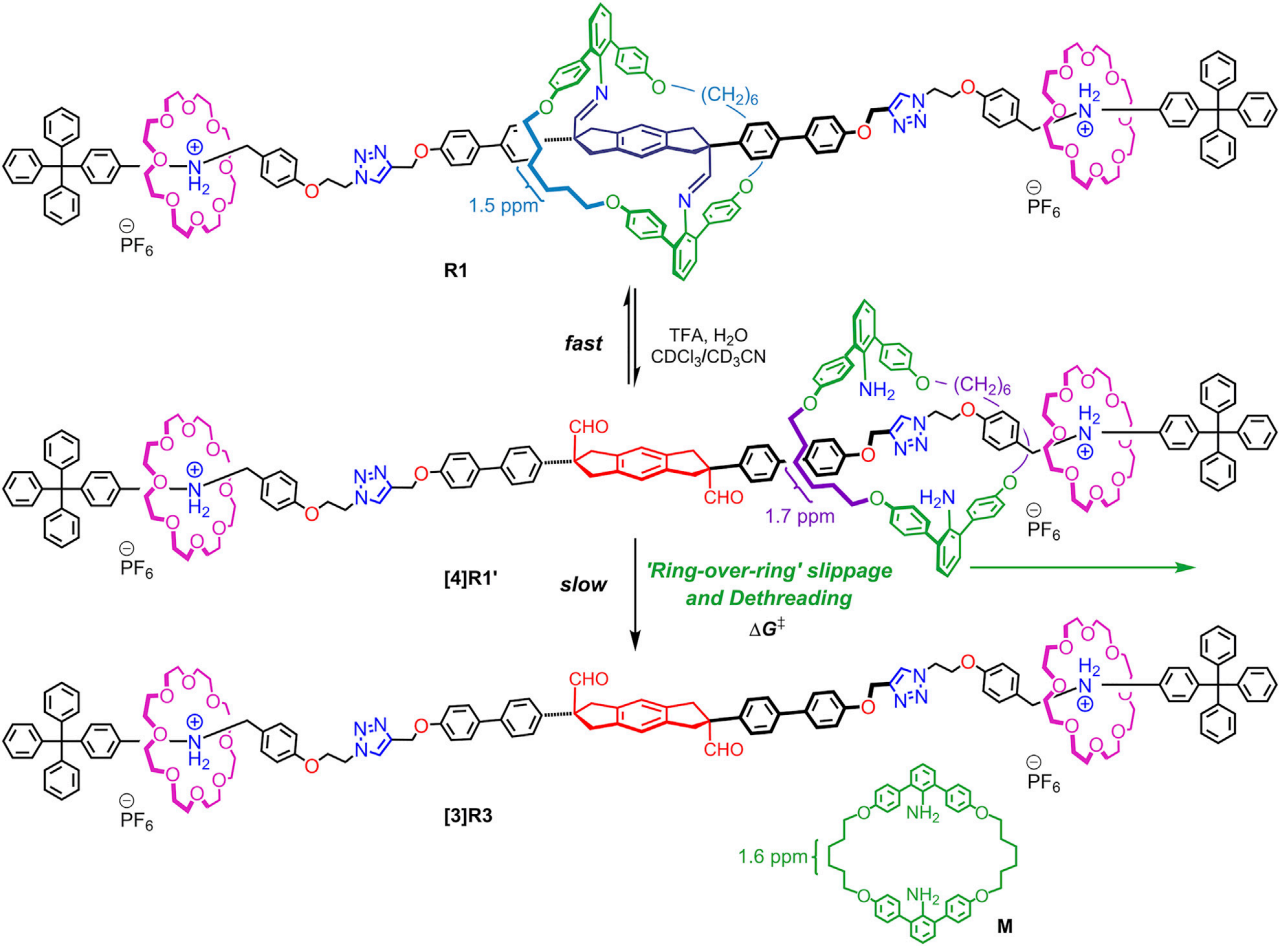
“Ring-over-ring” slippage and dethreading of macrocycle **M** after generation of hetero [4] rotaxanes **[4]R1′** from imine-bridged heterorotaxanes **R1**.

To observe this ring-over-ring slipping, we designed imine-bridged heterorotaxanes **R1** and **R2** as shown in Scheme 1. The key points are as follows: 1) the large aniline macrocycle (38-membered ring) is imine-bridged to the starting station to prevent it from falling off the end of the axle; 2) two crown ethers (24-membered rings) are located on the anilinium stations on both sides and are smaller than the central macrocycle; and 3) the triphenylmethyl end cap is large enough to prevent the crown ether from dethreading, but not to prevent the central macrocycle from dethreading. Therefore, when the imine bonds of this imine-bridged heterorotaxane **R1** are hydrolyzed to generate hetero[4]rotaxane **[4]R1′**, we expected that only the large macrocycle would be deslipped over the crown ether (ring-over-ring), producing the aniline macrocycle **M** and the [3]rotaxane **[3]R3** (Scheme 2). Here we report the synthesis of imine-bridged heterorotaxanes **R** and their deslipping behavior based on imine hydrolysis. Our results provide a new way to control the mobility and position of components in rotaxanes. Rotaxanes with multiple rings passing each other on a single track will open up new functionalities such as high flexibility and topological shape memory (Hart et al., 2021).

## Materials and Methods

^1^H and ^13^C NMR spectra were recorded on a Bruker BioSpin AVANCE DPX-400 and an AVANCE 400M (^1^H: 400 MHz, ^13^C: 100 MHz) spectrometer. IR spectra were taken on a JASCO FT/IR-4600 (ATR). HRMS analysis was performed on a JEOL JMS-S3000 SpiralTOF (MALDI-TOF). All melting points were determined on a METTLER TOLEDO MP90. Column chromatography was performed on silica gel 60 (YMC, particle size 40–63 μm). GPC purification was carried out on LC-908 with JAIGEL-1HH + 2HH columns eluted with CHCl_3_. Reactions were carried out under an argon atmosphere. All commercially available compounds were used without further purification unless otherwise indicated. The macrocycle **M** (Kawai et al., 2006), imine-bridged prerotaxane **P1** (Kawai et al., 2006), and 4-(2-bromoethoxy)benzaldehyde **E1** (Wang et al., 2015) were prepared by following the known procedures.

### Synthesis of N-[4-(2-Bromoethoxy) benzyl]-4-Tritylaniline E2

To a solution of **E1** (230 mg, 1.0 mmol) and 4-triphenylmethylaniline (350 mg, 1.0 mmol) in CHCl_3_ (55 mL) was added MS4Å. After the reaction mixture was stirred for 68 h at room temperature, it was filtered. The crude product obtained by concentrating the filtrate was diluted with THF (30 mL) and ethanol (30 mL), and NaBH_4_ (10.3 mg, 0.27 mmol) and anhydrous MgSO_4_ (ca. 50 mg) were added to it, stirred for 22 h at room temperature, and filtered. The filtrate was diluted with chloroform and water and separated. The organic layer was washed successively with H_2_O and brine, dried over MgSO_4_, and then filtered. The crude product obtained by concentrating the filtrate was subjected to chromatography on silica gel eluted with 12% ethyl acetate/hexane to give **E2** (185 mg, 34% yield, two steps) as a yellow solid.

M.p.: 102–150°C (decomp.); ^1^H NMR (400 MHz, CDCl_3_): δ/ppm 7.28 (d, *J* = 8.5 Hz, 2H), 7.25–7.16 (m, 15 H), 6.97 (d, *J* = 9.0 Hz, 2H), 6.88 (d, *J* = 9.0 Hz, 2H), 6.51 (d, *J* = 8.5 Hz, 2H), 4.28 (t, *J* = 6.3 Hz, 2H), 4.22 (s, 2H), 3.88 (s, 1H), 3.62 (t, *J* = 6.3 Hz, 2H); ^13^C NMR (100 MHz, CDCl_3_): δ/ppm 157.37, 147.29, 145.95, 135.90, 132.29, 132.01, 129.00, 127.29, 125.67, 114.91, 114.84, 111.75, 67.95, 64.21, 47.89, 29.07; IR (ATR): 3410, 3025, 2855, 1610, 1509, 1240, 1173, 819, 746, 699 cm^−1^; HR-MS (MALDI-TOF-MS, DCTB): Calcd. for C_34_H_30_BrNO+Na^+^: 570.1403, found: 570.1420.

### Synthesis of N-[4-(2-Azidoethoxy) benzyl]-4-Tritylaniline E3

To a solution of **E2** (710 mg, 1.3 mmol) in DMF (10 mL), was added NaN_3_ (84 mg, 1.3 mmol). The mixture was stirred at 100°C for 2 h. After the reaction mixture was cooled to room temperature, it was diluted with diethyl ether and washed successively with H_2_O and brine, dried over MgSO_4_, and then filtered. The yellow solid obtained by concentrating the filtrate was pure **E3** (642 mg, 97% yield).

M.p.: 146–154°C; ^1^H NMR (400 MHz, CDCl_3_): δ/ppm 7.29 (d, *J* = 8.5 Hz, 2H), 7.25–7.14 (m, 15 H), 6.98 (d, *J* = 9.0 Hz, 2H), 6.89 (d, *J* = 9.0 Hz, 2H), 6.52 (d, *J* = 8.5 Hz, 2H), 4.22 (s, 2H), 4.14 (t, *J* = 6.3 Hz, 2H), 3.88 (s, 1H), 3.58 (t, *J* = 6.3 Hz, 2H); ^13^C NMR (100 MHz, CDCl_3_): δ/ppm 157.1, 147.29, 145.96, 135.88, 132.20, 132.00, 131.13, 128.97, 127.29, 125.67, 114.73, 111.74, 67.01, 64.21, 50.15, 47.90; IR (ATR): 3384, 3053, 3029, 2931, 2843, 2088, 1610, 1510, 1240, 823, 746, 699, 629 cm^−1^; HR-MS (MALDI-TOF-MS, DCTB): Calcd. for C_34_H_30_N_4_O+Na^+^: 510.2414, found: 510.2435.

### Synthesis of N-[4-(2-Azidoethoxy) benzyl]-4-Tritylanilinium PF_6_ Salt E4

To a solution of **E3** (300 mg, 0.59 mmol) in methanol (9.0 mL) was added conc. HCl (0.1 mL). After the reaction mixture was stirred for 1 h at room temperature, it was filtered. The filtered ammonium salt was dissolved in a small amount of methanol to make a saturated solution, and satd. NH_4_PF_6_aq. was added until no more solid precipitated. The yellow solid obtained by filtering the suspension was pure **E4** (360 mg, 93% yield).

M.p.: 128–136°C; ^1^H NMR (400 MHz, CDCl_3_): δ/ppm 7.31–7.08 (m, 17H), 7.02 (d, *J* = 8.6 Hz, 2H), 6.97 (d, *J* = 8.6 Hz, 2H), 6.80 (d, *J* = 8.6 Hz, 2H), 4.54 (s, 2H), 4.10 (t, *J* = 5.0 Hz, 2H), 3.59 (t, *J* = 5.0 Hz, 2H), 1.55 (br.s, 2H); ^13^C NMR (400 MHz, CDCl_3_): δ/ppm 159.28, 159.20, 145.92, 132.55, 132.46, 130.93, 127.70, 126.28, 122.68, 114.69, 97.09, 66.88, 64.74, 50.06; IR (ATR): 3162, 2935, 2117, 1611, 1516, 1255, 1180, 820, 748, 702, 633, 556 cm^−1^.

### Synthesis of Imine-Bridged Prerotaxane P2

To a solution of **P1** (123 mg, 80 μmol) in dry DMF (8 mL) and dry THF (12 mL) at room temperature, was added TBAF (1.0 M solution in THF, 190 μL, 190 μmol) under an argon atmosphere. After the mixture was stirred for 10 min, Cs_2_CO_3_ (130 mg, 0.4 mmol) and 3-bromopropyne (57 μL, 0.77 mmol) were added. The mixture was stirred for 16 h at room temperature. The reaction mixture was diluted with CH_2_Cl_2_ and washed successively with satd. NH_4_Claq., H_2_O, and brine, dried over MgSO_4_, and then filtered. The crude product obtained by concentrating the filtrate was purified by GPC separation to give **P2** (56 mg, 51%) as a white solid.

M.p.: 201–250°C (decomp); ^1^H NMR (400 MHz, CDCl_3_): δ/ppm 7.47 (d, *J* = 8.5 Hz, 4H), 7.42 (d, *J* = 8.5 Hz, 4H), 7.32–6.52 (m, 30H), 4.73 (s, 4H), 4.22–3.96 (m, 8H), 3.24 (d, *J* = 15 Hz, 4H), 3.10 (d, *J* = 15 Hz, 4H), 2.54 (t, *J* = 2.1 Hz, 4H), 1.92–1.76 (m, 8H), 1.57–1.48 (m, 8H), 1.26 (s, 2H); ^13^C NMR (100 MHz, CDCl_3_): δ/ppm 169.34, 157.04, 148.60, 143.02, 139.24, 139.06, 134.11, 133.36, 132.35, 131.01, 130.80, 129.03, 128.58, 128.21, 128.07, 127.61, 126.82, 125.29, 123.88, 120.62, 115.22, 114.82, 114.54, 78.51, 75.61, 68.25, 56.55, 55.88, 40.45, 25.87, 25.75, 25.60; IR (ATR): 3250, 3030, 3007, 2925, 2858, 1725, 1649, 1606, 1510, 1496, 1235, 1174, 1017, 1001, 821, 795, 751 cm^−1^; HR-MS (MALDI-TOF-MS, DHB): Calcd. for C_92_H_80_N_2_O_6_+H^+^: 1309.6089, found: 1309.6142.

### Synthesis of Imine-Bridged Heterorotaxane R1

Pseudorotaxane with a [24]C8 was prepared by adding [24]C8 (21 mg, 57 μmol) to **E4** (19 mg, 29 μmol) in dry CH_2_Cl_2_ (1.2 mL) and stirring at room temperature for 10 min. Under an argon atmosphere **P2** (15 mg, 11 μmol) and [Cu(MeCN)_4_]PF_6_ (10 mg, 29 μmol) were added to the solution. The mixture was stirred for 6 h at room temperature in the dark and concentrated. The crude product was washed with methanol to give **R1** (28 mg, 73%) as a pale brown solid.

M.p.: 162–170°C (decomp); ^1^H NMR (400 MHz, CD_3_CN/CDCl_3_ = 1:1): δ/ppm 8.99 (br.s, 4H), 7.96 (br.s, 2H), 7.48–6.45 (m, 86H), 5.22 (s, 4H), 5.07–4.99 (m, 4H), 4.80 (t, *J* = 4.6 Hz, 4H), 4.45 (t, *J* = 4.6 Hz, 4H), 4.17–4.00 (m, 8H), 3.49–3.20 (m, 64H), 3.12 (d, *J* = 15.0 Hz, 4H), 1.91–1.76 (m, 8H), 1.61–1.45 (m, 8H); ^13^C NMR (100 MHz, CD_3_CN/CDCl_3_ = 1:1): δ/ppm 170.14, 159.28, 158.52, 157.67, 149.44, 148.86, 146.71, 143.38, 139.75, 139.26, 136.16, 133.84, 133.79, 133.67, 132.58, 132.51, 131.44, 132.29, 131.37, 131.17, 129.02, 128.46, 128.39, 128.20, 127.91, 127.09, 126.76, 125.96, 124.45, 122.64, 120.95, 115.66, 115.24, 80.65, 70.86, 70.72, 68.57, 66.97, 65.44, 62.18, 57.64, 51.09, 50.16, 40.94, 29.35, 26.18; IR (ATR): 2870, 2357, 1607, 1511, 1496, 1457, 1350, 1238, 1177, 1091, 1033, 955, 837, 749, 702, 556 cm^−1^; HR-MS (MALDI-TOF-MS, DHB): Calcd. for C_192_H_205_N_10_O_24_^+^+H_2_O: 3053.5307, found: 3053.539.

### Synthesis of Imine-Bridged Heterorotaxane R2

Pseudorotaxane with a DB[24]C8 was prepared by adding DB[24]C8 (13 mg, 29 μmol) to **E4** (19 mg, 29 μmol) in dry CH_2_Cl_2_ (0.6 mL) and stirring at room temperature for 10 min. Under an argon atmosphere **P2** (7.5 mg, 6 μmol) and [Cu(MeCN)_4_]PF_6_ (5.0 mg, 15 μmol) were added to the solution. The mixture was stirred for 6 h at room temperature in the dark and concentrated. The crude product was washed with hot toluene to give **R2** (6.5 mg, 36%) as a pale brown solid.

M.p.: 170–195°C (decomp); ^1^H NMR (400 MHz, CD_3_CN/CDCl_3_ = 1:1): δ/ppm 8.95 (br.s, 4H), 7.96 (br.s, 2H), 7.52–6.60 (m, 94H), 5.24 (s, 4H), 5.19–5.12 (m, 4H), 4.73 (t, *J* = 4.5 Hz, 4H), 4.24 (t, *J* = 4.5 Hz, 4H), 4.08 (m, 9H), 3.98–3.93 (m, 18H), 3.64 (s, 16H), 3.32–3.16 (m, 18H), 3.13 (d, *J* = 15.0 Hz, 4H) 1.90–1.73 (m, 8H), 1.60–1.41 (m, 8H); ^13^C NMR (100 MHz, CD_3_CN/CDCl_3_ = 1:1): δ/ppm 158.94, 149.12, 147.59, 147.57, 146.64, 133.29, 132.28, 131.37, 131.29, 131.22, 131.17, 128.65, 128.54, 128.51, 128.47, 128.31, 127.55, 126.72, 125.55, 125.52, 122.03, 121.75, 121.13, 115.73, 115.69, 115.47, 115.26, 114.99, 112.49, 70.97, 70.39, 68.32, 66.75, 65.25, 51.37, 50.18, 50.16, 38.90, 28.97; IR (ATR): 2936, 2363, 1506, 1247, 1123, 836, 744, 555 cm^−1^; HR-MS (MALDI-TOF-MS, DHB): Calcd. for C_208_H_205_N_10_O_24_^+^: 3226.5113, found: 3226.5068.

### Deslipping of Macrocycle from Imine-Bridged Heterorotaxanes R1 and R2

To a solution of imine-bridged heterorotaxanes **R1** (0.50 mg, 0.30 μmol) or R2 (0.25 mg, 0.15 μmol) in water-saturated CDCl_3_ and CD_3_CN (v/v 1:1, 0.5 mL) in an NMR tube was added 10% TFA in CDCl_3_ (100 eq.). The NMR tube was kept at constant temperature in a thermostatic bath at 30, 40, or 50°C. The time-courses of hydrolysis and attainment of equilibrium to give macrocycle **M** and **[3]R3** or **[3]R4** were monitored by ^1^H NMR spectroscopy.

[3]rotaxane **[3]R3**: ^1^H NMR (400 MHz, CD_3_CN/CDCl_3_ = 1:1): δ/ppm 9.48 (s, 2H), 9.00 (br.s, 4H), 8.02 (s, 2H), 7.66–6.54 (m, 66H), 5.22 (s, 4H), 5.25 (s, 4H) 5.03 (br.s, 4H), 4.83 (t, *J* = 5.0 Hz, 4H), 4.47 (t, *J* = 5.0 Hz, 4H), 3.81 (d, *J* = 15 Hz, 4H), 3.34–3.24 (m, 68H).

[3]rotaxane **[3]R4**: ^1^H NMR (400 MHz, CD_3_CN/CDCl_3_ = 1:1): δ/ppm 9.47 (s, 2H), 8.97 (br.s, 4H), 7.94 (s, 2H), 7.66–6.54 (m, 82H), 5.22 (s, 4H), 5.21–5.14 (m, 4H), 4.74 (t, *J* = 5.0 Hz, 4H), 4.25 (t, *J* = 5.0 Hz, 4H), 4.14–3.73 (m, 18H), 3.80 (d, *J* = 15 Hz, 4H), 3.66 (s, 16H), 3.38–3.18 (m, 22H).

## Results and Discussion

### Synthesis and Characterization

**SCHEME 3:**
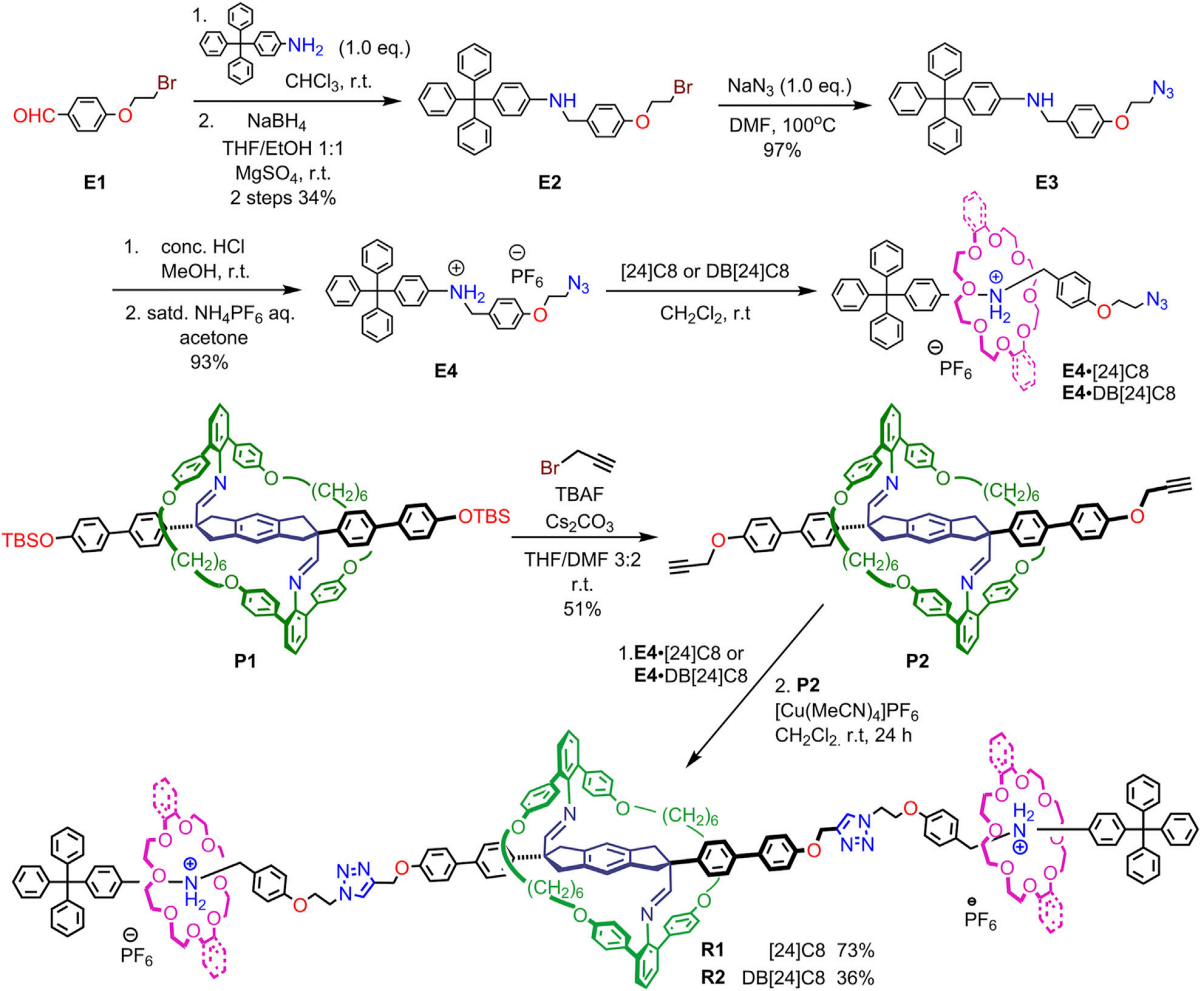
Preparation of imine-bridged heterorotaxanes **R1** and **R2**.

The synthesis of imine-bridged heterorotaxanes **R1** and **R2** with one 38-membered aniline macrocycle and two [24]crown-8 ([24]C8) or dibenzo[24]crown-8 (DB24C8) rings on the axle is shown in Scheme 3. Pseudorotaxane **E4**•[24]C8 was prepared by threading [24]C8 added to a triphenylmethyl-type end cap **E4** containing an anilinium station for the crown ether and terminated with an azide group in CH_2_Cl_2_ solution. The desired imine-bridged heterorotaxane **[3]R1** was obtained by the CuAAC reaction in 73% yield by adding 0.5 equivalents of propargyl-terminated imine-bridged prerotaxane **P2** and Cu (MeCN)_4_PF_6_ to the prepared solution of pseudorotaxane **E4**•DB[24]C8. Imine-bridged heterorotaxane **R2** with DB[24]C8 was prepared by the same method in 36% yield.

**FIGURE 3:**
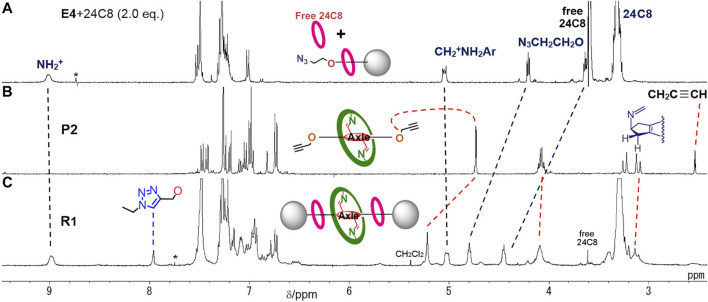
^1^H NMR spectra (400 MHz, at 298 K) of **(A)** pseudorotaxane **E4**•[24]C8 and free [24]C8 in CDCl_3_, **(B)** imine-bridged prerotaxane **P2** in CDCl_3_, and **(C)** imine-bridged heterorotaxane **R1** in CDCl_3_/CD_3_CN. *: impurity or satellite from solvent peak.

**FIGURE 4:**
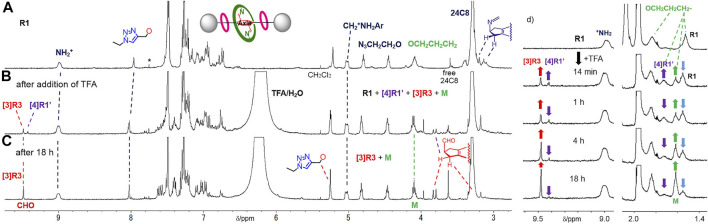
^1^H NMR spectra (400 MHz, CDCl_3_/CD_3_CN at 303 K) of **(A)** imine-bridged heterorotaxane **R1**, **(B)** hetero[4]rotaxane **[4]R1′** and [3]rotaxane **[3]R3** generated upon the addition of TFA to a solution of **R1**, **(C)** [3]rotaxane **[3]R3** and macrocycle **M** after 18 h and **(D)** time-course of hetero[4]rotaxane **[4]R1′** and dethreaded macrocycle **M** from imine-bridged heterorotaxane **R1** upon imine hydrolysis. *: impurity or satellite from solvent peak.

The rotaxane moiety with [24]C8 was evidenced by the appearance of benzylanilinium CH_2_ and ^+^NH_2_ moieties at large downfield shifted positions of 5.0 and 9.0 ppm, which were similar to the values observed for other rotaxanes with [24]C8 (Kimura et al., 2017) (Figure 3). In addition, the threaded [24]C8 part appeared as a large singlet at 3.3 ppm, which overlapped with the signal of the central imine-bridged station. The characteristic feature of the imine-bridged station of **R1** is the methylene moiety of the five-membered ring appearing as two sets of doublets at around 3.2 ppm, which can be compared with the sets at 3.2 and 3.8 ppm of the bisaldehyde station resulting from the hydrolysis of the imine bonds (Kawai et al., 2006). Furthermore, the signals of the OCH_2_CH_2_CH_2_- moiety of the aniline macrocycle appear at 4.1, 1.8, and 1.5 ppm, which are close to those at 4.1, 1.8, and 1.6 ppm of the macrocycle **M** itself (Figure 4D). These peaks can be used as one of the probes to study the deslipping of the aniline macrocycle **M**. These observations confirm that the large aniline macrocycle is located on the imine station and the two [24]C8s are located on the benzyl anilinium stations at both ends, and therefore the heterorotaxane structure is stable without any dethreading of either ring.

### Ring-Over-Ring Deslipping

Next, we investigated whether the aniline macrocycle by hydrolyzing imine bonds of **R1** could be deslipped over the small ring and end cap at either end to dissociate into hetero[4]rotaxane **[4]R1′** and macrocycle **M** (Scheme 3). It was observed that when TFA was added to a CDCl_3_/CD_3_CN 1:1 (v/v) solution of imine-bridged heterorotaxane **R1**, several chemical species immediately appeared, some of which increased with time (Figure 4). The hydrolysis of the imine bonds was confirmed by the two aldehyde signals appearing at around 9.5 ppm and the doublet at 3.8 ppm of the five-membered ring proton at the central station, which were also observed to increase with time, and the signals of imine-bridged **R1** almost disappeared after 24 h. The rotaxane structure with [24]C8 remained unchanged during these processes, which was confirmed by the fact that the signals at these sites remained constant in position and intensity. Most importantly, the increase in the signal at 1.6 ppm, which is characteristic of macrocycle **M** itself, demonstrates the ‘ring-over-ring’ deslipping of the aniline macrocycle **M** over [24]C8, accompanying the hydrolysis of the imine bonds.

**FIGURE 5:**
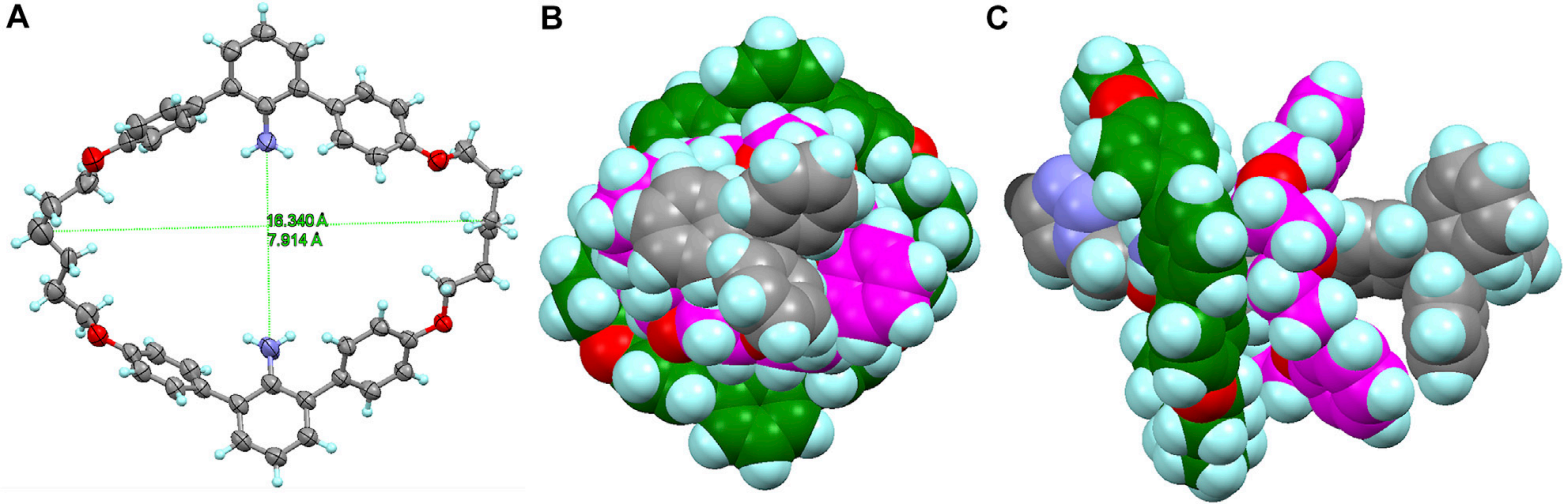
**(A)** X-ray structure of macrocycle **M** (CCDC 2158687). **(B)** Front view and **(C)** top view of the molecular model of macrocycle **M** and DB24C8 on the axle moiety of **[4]R2′** (terminated with triazole). The molecular model (neither the most stable structure nor the transition structure) was prepared by using the SPARTAN ’14 to estimate the size of the rings.

An interesting finding was the observation of hydrolyzed hetero[4]rotaxane **[4]R1′** as an intermediate in the process of deslipping, which provides important insight into the mechanism (rate-limiting step) of deslipping. Interestingly, the signal of the macrocycle CH_2_- moiety in the hydrolyzed hetero[4]rotaxane **[4]R1′** was observed at 1.7 ppm, which remained almost constant in intensity as a steady state and finally disappeared. Also in the aldehyde station of the axle molecule, the signal of the intermediate appeared as a steady state at 9.4 ppm and finally disappeared with the consumption of the imine form **R1**. This result implies that the hydrolysis of the imine bond of **R1** proceeds fast, resulting in a dynamic equilibrium state with the hetero[4]rotaxane **[4]R1′**, from which the deslipping of macrocycle M is a much slower, rate-limiting step. This is also consistent with previous results where an imine-bridged rotaxane immediately produces hydrolyzed [2]rotaxane upon addition of acid, resulting in a dynamic equilibrium (Kawai et al., 2006). It has also been shown that the presence of an additional hydrogen bonding station biases the equilibrium ratio toward the hydrolyzed rotaxane (the ratio of hydrolyzed rotaxane in CDCl_3_ at room temperature is 9% without a hydrogen bonding station and 95% with a TEG station) (Umehara et al., 2008). During the hydrolysis of **R1** in this study, the ratio of imine-bridged heterorotaxane **R1** to the intermediate hydrolyzed hetero[4]rotaxane **[4]R1′** was about 2:1 (Figure 5), suggesting that the aniline macrocycle weakly interacts with the crown ether or triazole moiety in the hydrolyzed hetero[4]rotaxane **[4]R1′**. These results suggest that the rate-limiting step of deslipping is not the hydrolysis of the imine bonds of **R1**, but the deslipping step of the aniline macrocycle from the hydrolyzed hetero[4]rotaxane **[4]R1′**.

Furthermore, the deslipping seems to be irreversible under these conditions, since the starting material **R1** was almost completely consumed and the imine form **R1** was not regenerated from the resulting [3]rotaxane **[3]R3** and macrocycle **M**. A comparable deslipping study was also performed using imine-bridged heterorotaxane **R2** containing the bulkier DB[24]C8 (Supplementary Figure S13, See Supplementary Material). The deslipping proceeded, although it required a longer time (τ_1/2_ = 27.2 h) than that of **R1** (τ_1/2_ = 9.2 h), confirming that the aniline macrocycle was large enough to overcome DB[24]C8 (Figure 5; Supplementary Figure S14).

**FIGURE 6:**
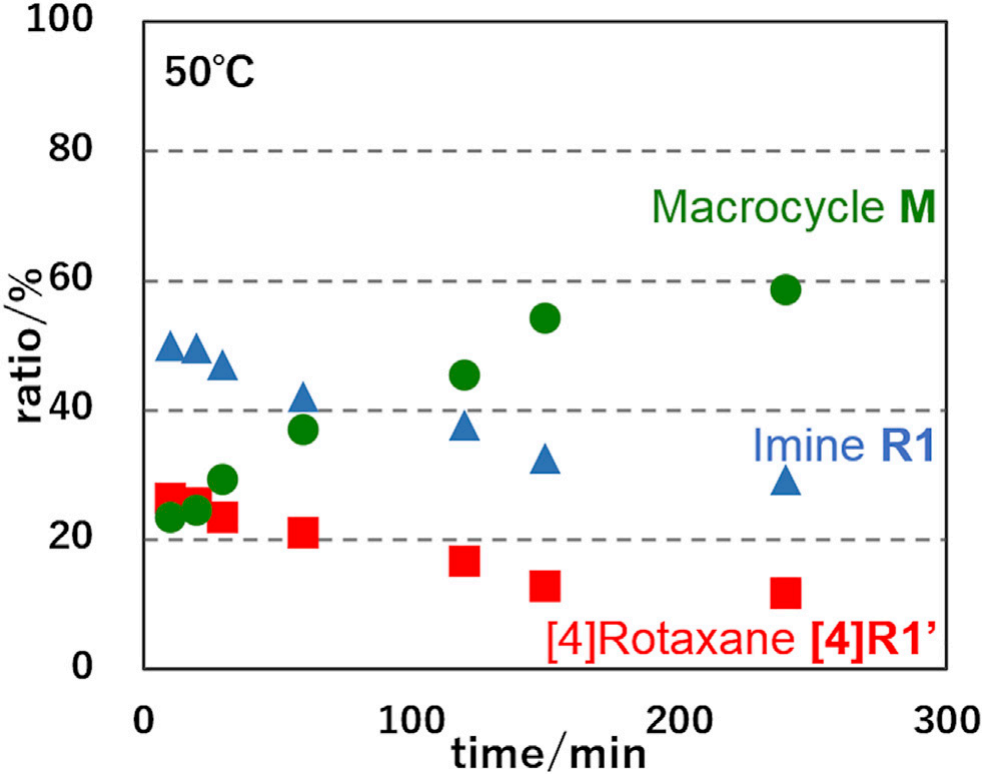
Time-courses of ratios of imine-bridged heterorotaxane **R1**, hetero[4]rotaxane **[4]R1′** and dethreaded macrocycle **M** upon imine hydrolysis of **R1** at 323 K.

In order to determine the thermodynamic parameters of this deslipping process, we monitored the deslipping reaction at 303, 313, and 323 K (Figure 6; Supplementary Figure S15) and performed a pseudo-first order reaction kinetics analysis as the dissociation from the [4]rotaxane components (sum of imine **R1** and hetero[4]rotaxane **[4]R1′**) to [3]rotaxane **[3]R3** and macrocycle **M**. The results are shown in Table 1. The thermodynamic parameters of **R1** with [24]C8 at 303 K were determined to be a rate constant *k* of 0.94 × 10^−3^ s^−1^ and half-life of 9.2 h (Supplementary Figure S16). The Δ*H* ^‡^, Δ*S* ^‡^, and Δ*G*_303_^‡^ values in the transition state are 12.0 kcal mol^−1^, -33.1 calmol^−1^K^−1^, and 22.0 kcal mol^−1^, respectively, indicating that the aniline macrocycle is sterically hampered by the crown ether moiety and its mobility is severely limited in the transition state. In **R2** with DB[24]C8, the Δ*G*_303_^‡^ is 22.3 kcal mol^−1^ and the half-life is 27.2 h, reflecting the increased steric hindrance compared to **R1**. Comparison of these parameters for the deslipping of the 38-membered macrocycle **M** from **[4]R1′** with those of the 42-membered ring B42C8 of Loeb’s system to pass over the same 24C8 ring (Zhu et al., 2018) would provide important insight into the understanding of the dynamics of mechanically bonded molecules. Loeb et al. discussed the extra energy cost required for the “ring-through-ring” slipping based on the difference in shuttling parameters between [2]rotaxane and hetero[3]rotaxane (Δ*H* ^‡^ = +1.6 kcal mol^−1^, Δ*S* ^‡^ = −4.8 cal mol^−1^K^−1^). The higher enthalpy and entropy costs for **[4]R1′** (Δ*H* ^‡^ = 12.0 kcal mol^−1^ and Δ*S* ^‡^ = −33.1 cal mol^−1^K^−1^) compared to Loeb’s rotaxane is presumably due to the larger steric hindrance due to the smaller inner cavity of the 38-membered macrocycle **M** with inner amino groups (Figure 6), the flexibility based on two hexamethylene linkers, and the weaker interaction with the rotaxane axle.

**TABLE 1:** Thermodynamic parameters for the “ring-over-ring” dethreading macrocycle **M** from hetero[4]rotaxanes **R1** and **R2** to give [3]rotaxanes **[3]R3** and **[3]R4** in CDCl_3_/CD_3_CN.

	Temp. K	*k* s^−1^	*τ*_1/2_ h	Δ*G* ^‡^ kcal mol^−1^	Δ*H* ^‡^ kcal mol^−1^	Δ*S* ^‡^ cal mol^−1^ K^−1^
**R1**	323	3.46 × 10^−3^	2.2	22.7	12.0	33.1
	313	1.33 × 10^−3^	5.8	22.3		
	303	0.94 × 10^−3^	9.2	20.0		
**R2**	303	0.49 × 10^−3^	27.2	22.3	-	-

## Conclusion

In summary, we constructed imine-bridged heterorotaxanes **R1** and **R2** with two different sized rings to investigate the rings passing each other on the rotaxane axle (single track). When the imine bridges of **R1** were cleaved, a hydrolyzed hetero[4]rotaxane **[4]R1′** was generated as an intermediate under dynamic equilibrium, and finally the large aniline macrocycle was deslipped over the crown ether to dissociate into a [3]rotaxane **[3]R3** and a macrocycle **M**. The determined thermodynamic parameters revealed that the rate-limiting step of the deslipping process was attributed to steric hindrance between two rings and reduced mobility due to the proximity of **M** to the crown ether, which was bound to the anilinium on the axle molecule. For the future construction and operation of molecular machines, it is desirable to control the motion and state of multiple different components independently. The “ring-through-ring” mobility on the axle adds a new dimension to the motion control of rotaxanes as well as challenges for how to deal with interactions and steric hindrance between components. We are currently working on the development of a ratcheting function by combining ring-through-ring slippage with gating control of imine bridges.

## Data Availability

The original contributions presented in the study are included in the article/Supplementary Material, further inquiries can be directed to the corresponding author.
