# Supplementary material for: Ring-Over-Ring Deslipping From Imine-Bridged Heterorotaxanes
Source: Front Chem. 2022 May 3;10:885939. doi: 10.3389/fchem.2022.885939 (PMC9110657; doi:10.3389/fchem.2022.885939)
Supplement: Supplementary file 1 [file DataSheet1.PDF]

Supplementary Material

Ring-over-ring Deslipping from Imine-Bridged Heterorotaxanes

Sayaka Hoshino, Kosuke Ono and Hidetoshi Kawai*

Contents

1. ^{1}H and ^{13}C NMR Spectra of Novel Compounds S2-S7

2. Deslipping of Macrocycle from Imine-Bridged Heterorotaxane **R2** S8

3. Single Crystal X-ray Structural Analysis of Macrocycle **M** S9

4. Molecular Models of Macrocycle, Crown Ether and Endcap Moieties S10

5. Kinetics and Thermodynamic Data for 'Ring-over-Ring' Deslipping S11-S12

1. ^{1}H and ^{13}C NMR Spectra of Novel Compounds

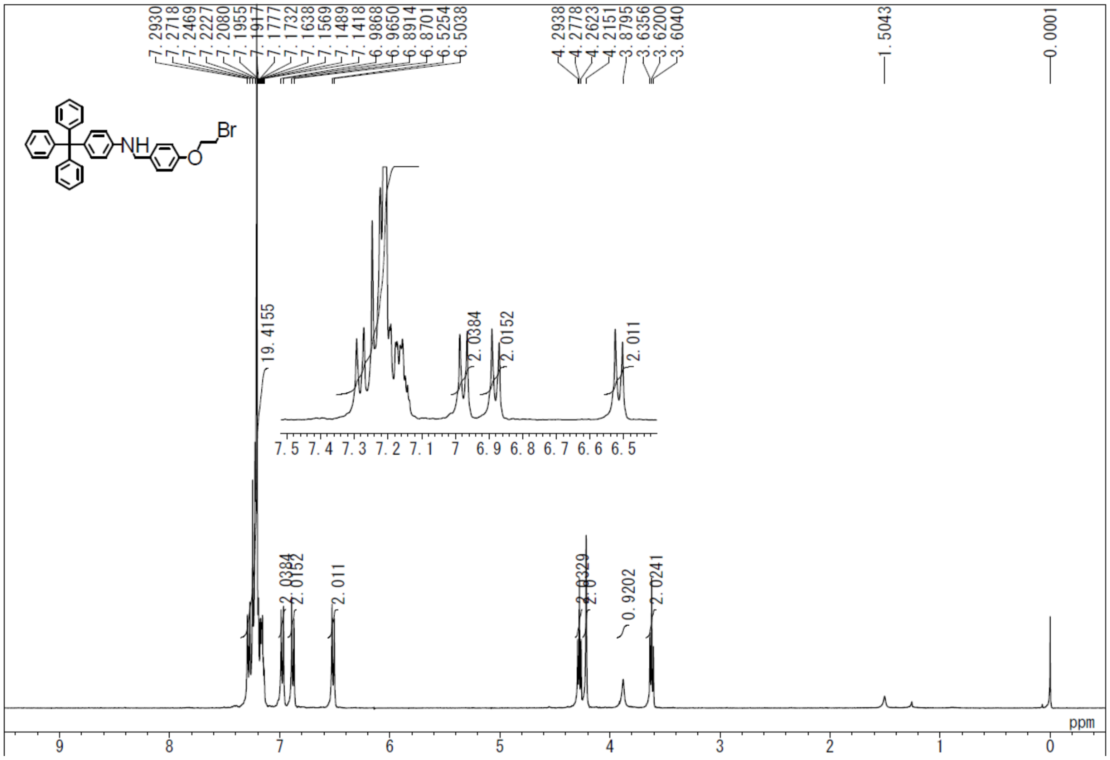

Figure S1. ^{1}H-NMR spectrum of **E2**.

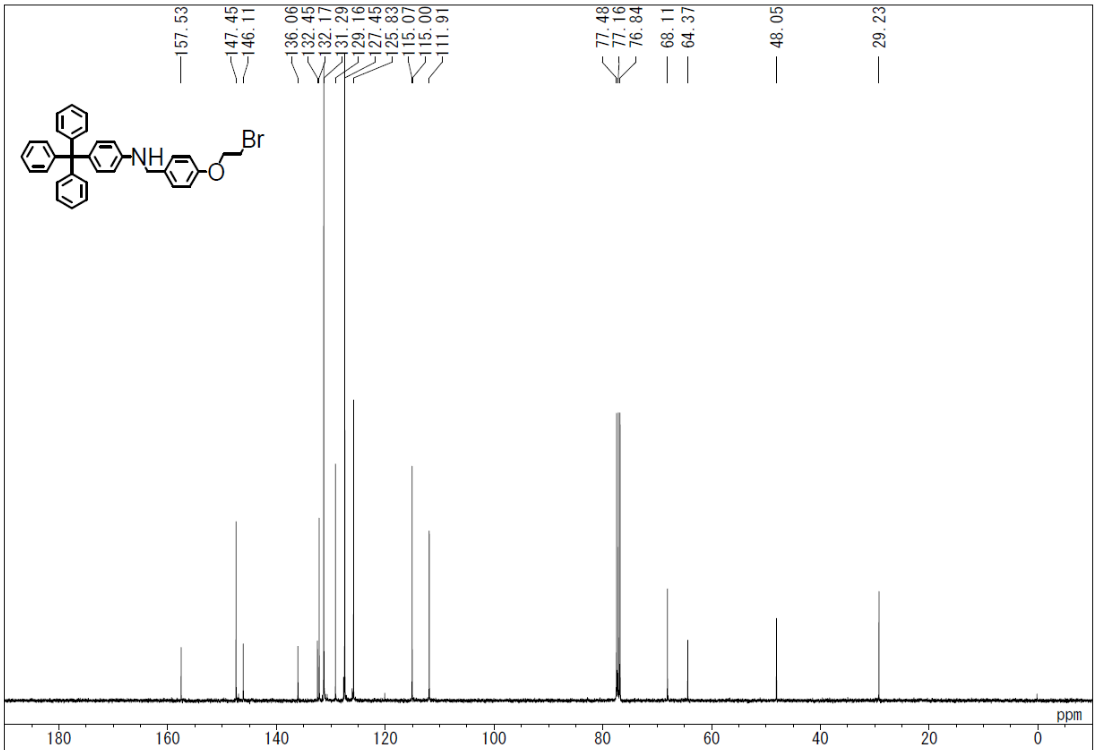

Figure S2. ^{13}C-NMR spectrum of **E2**.

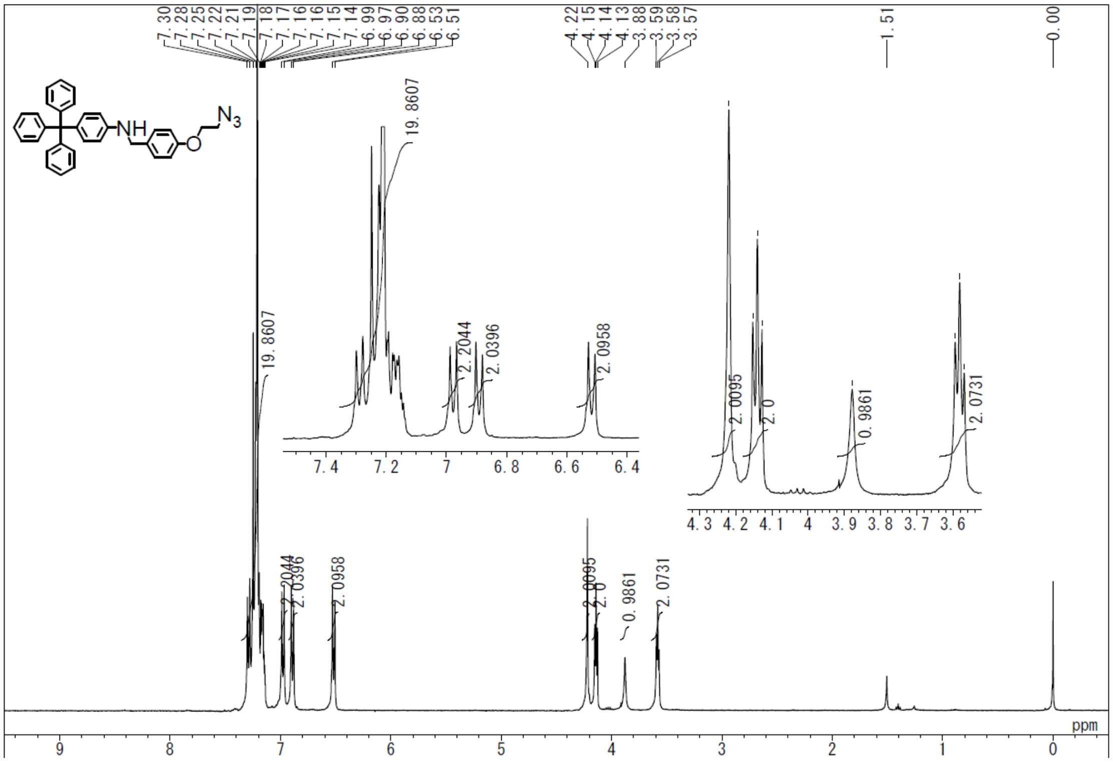

Figure S3. ^{1}H-NMR spectrum of **E3**.

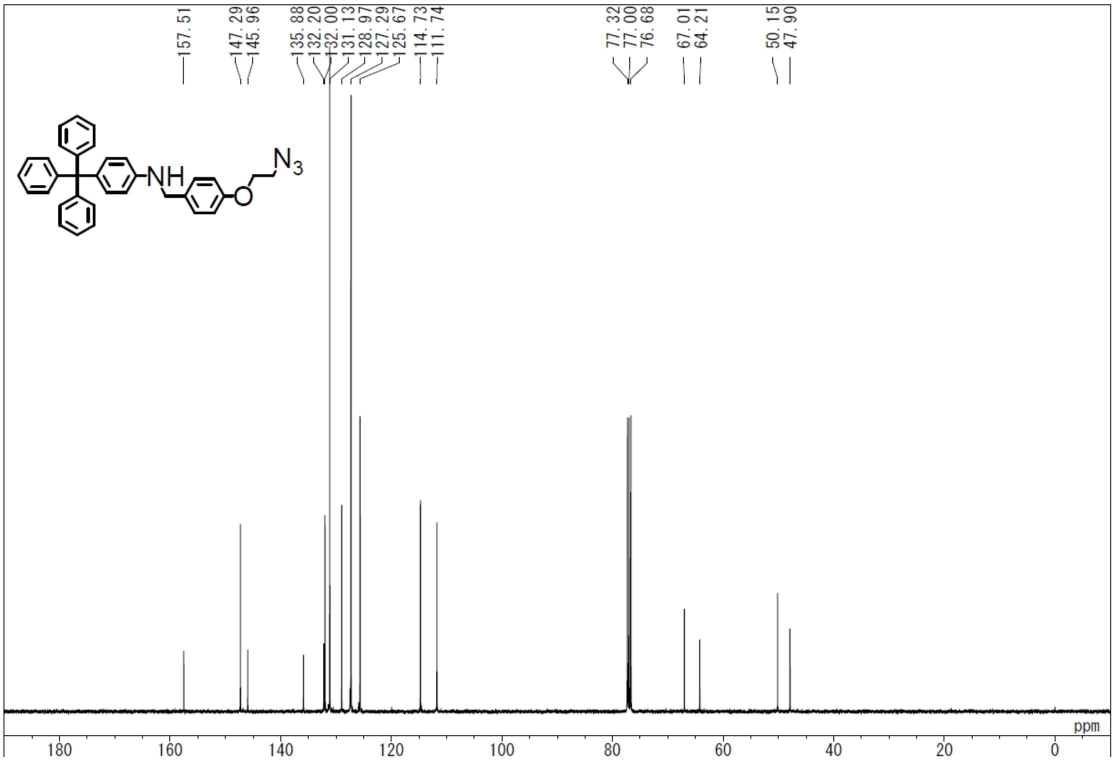

Figure S4. ^{13}C-NMR spectrum of **E3**.

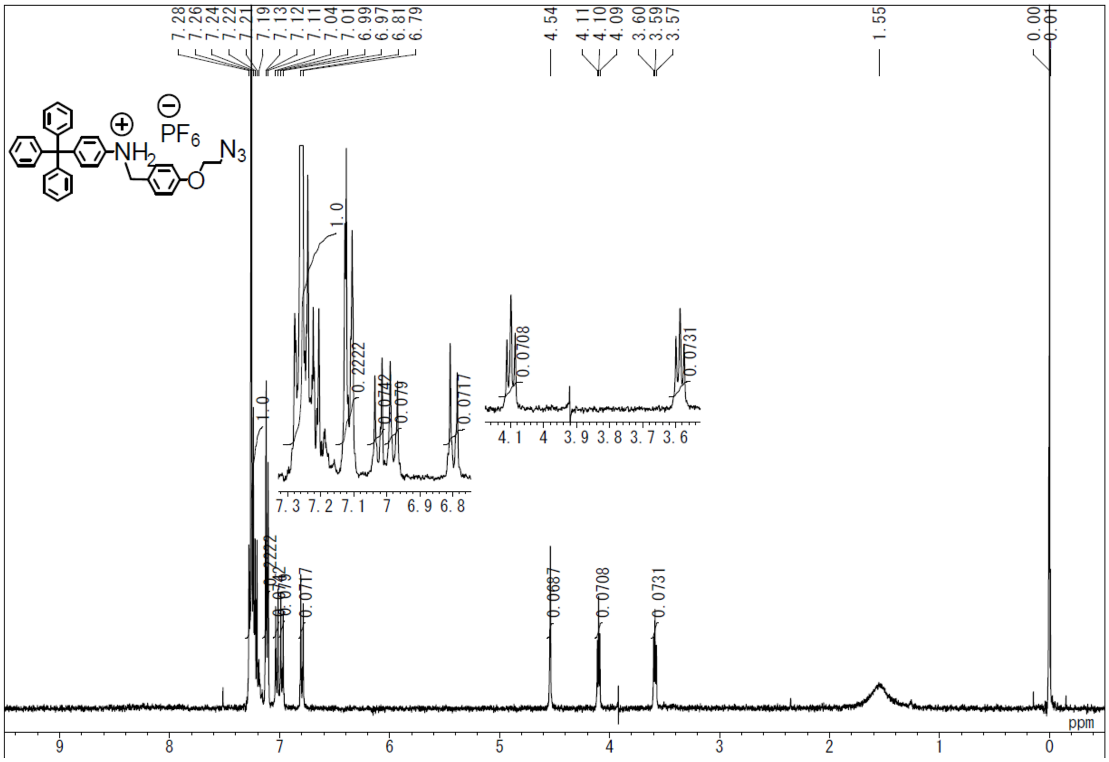

Figure S5. ^{1}H-NMR spectrum of **E4**.

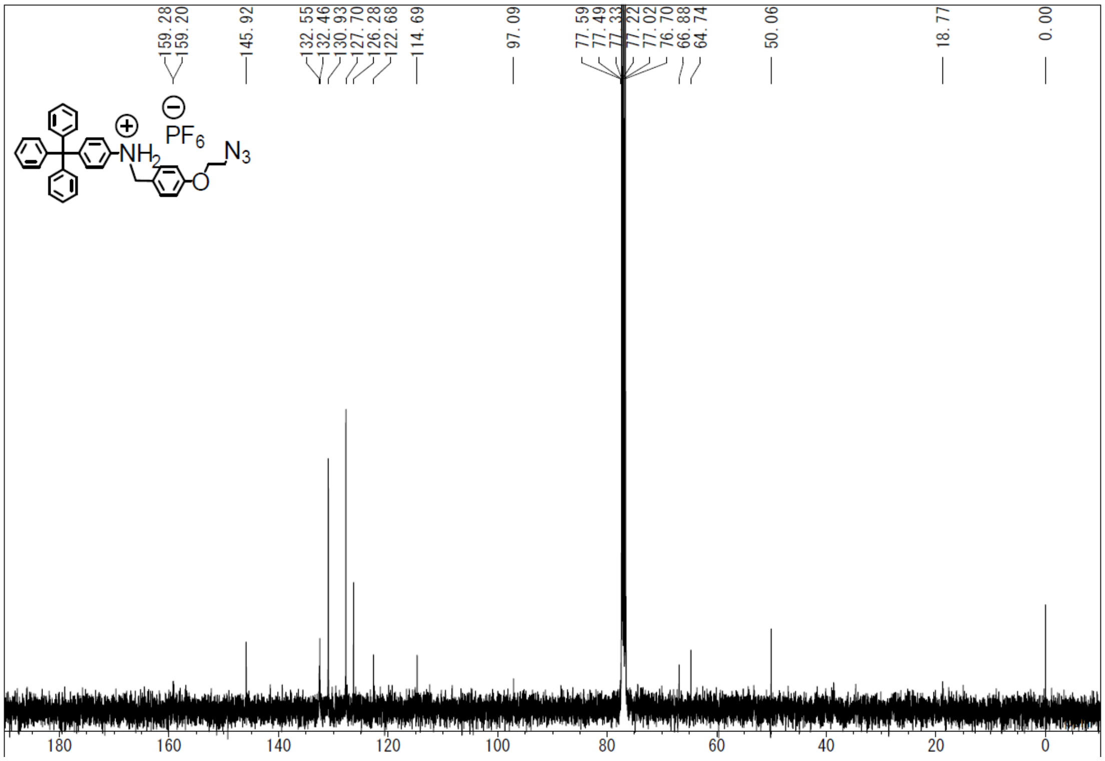

Figure S6. ^{13}C-NMR spectrum of **E4**.

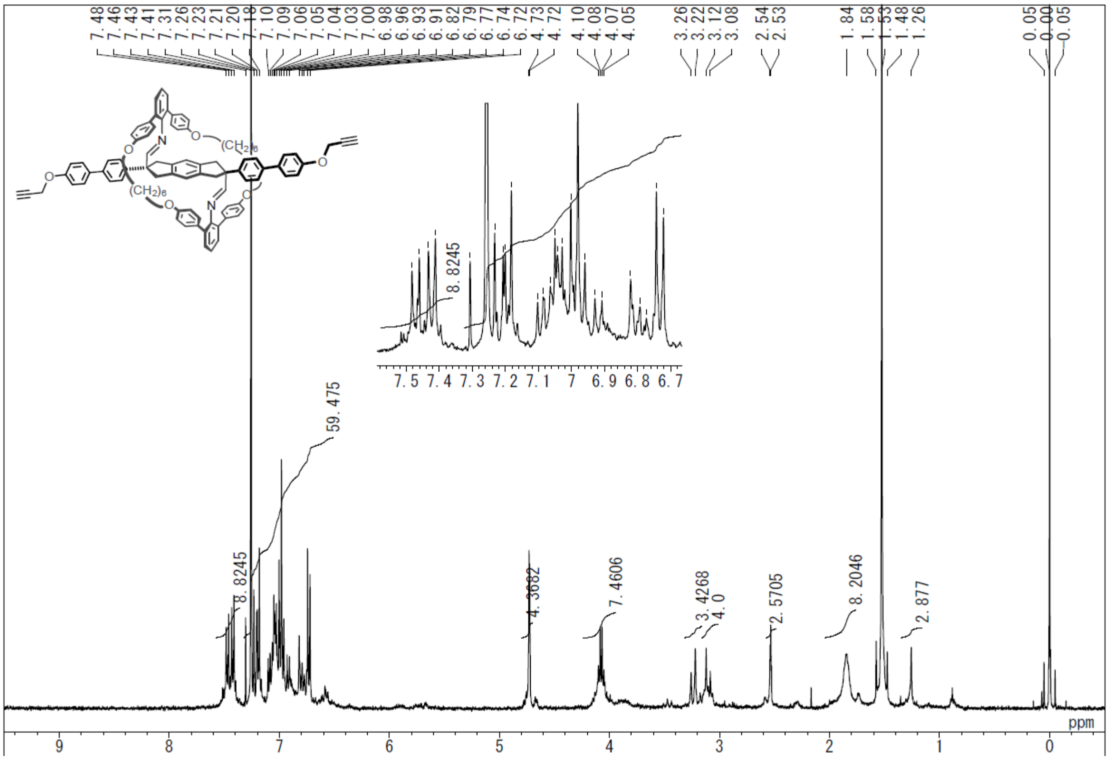

Figure S7. ^{1}H-NMR spectrum of **P2**.

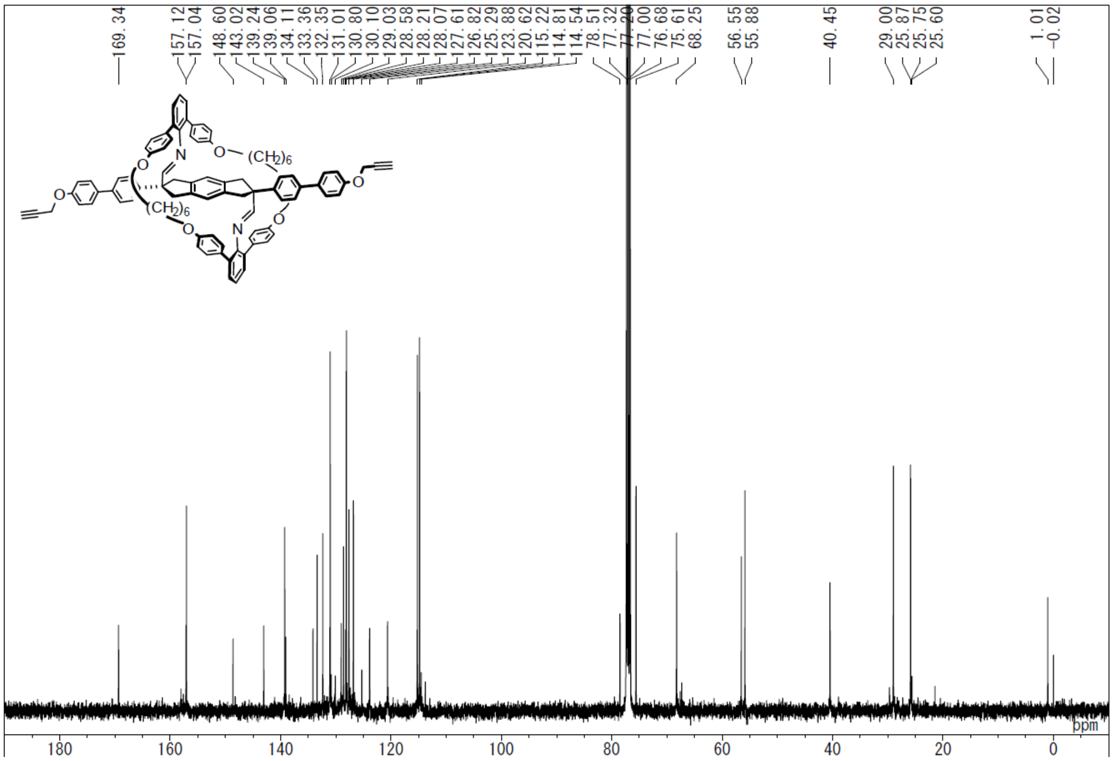

Figure S8. ^{13}C-NMR spectrum of **P2**.

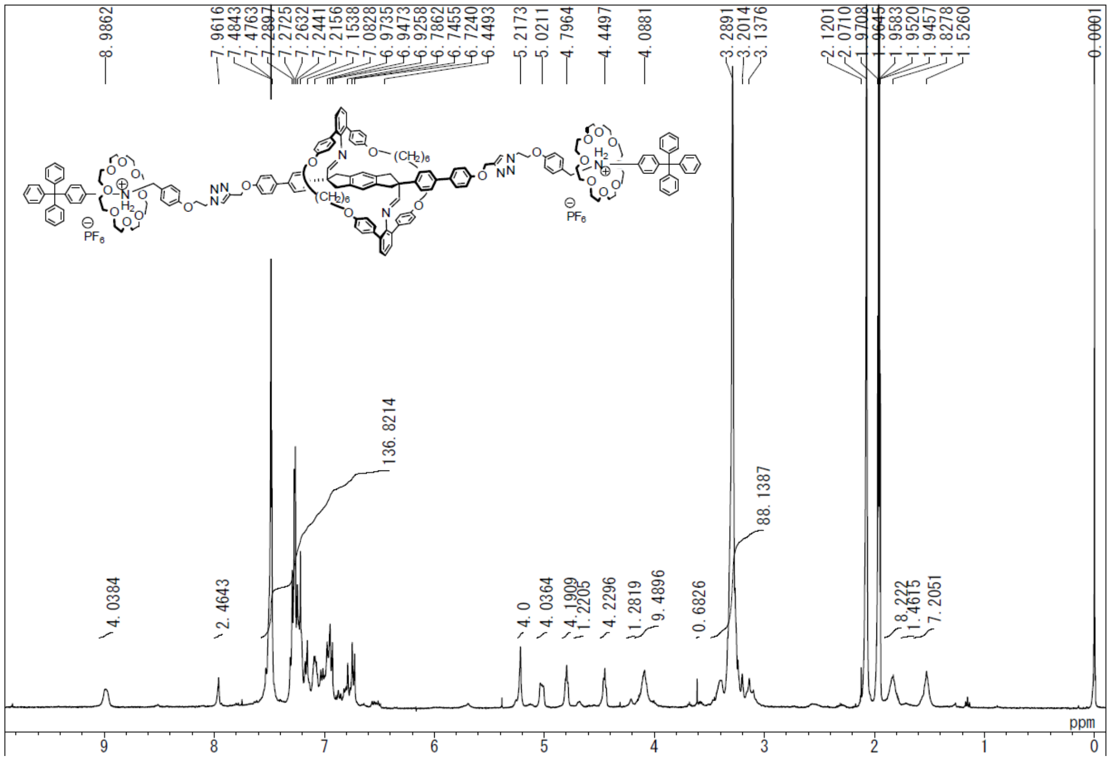

Figure S9. ^{1}H-NMR spectrum of **R1**.

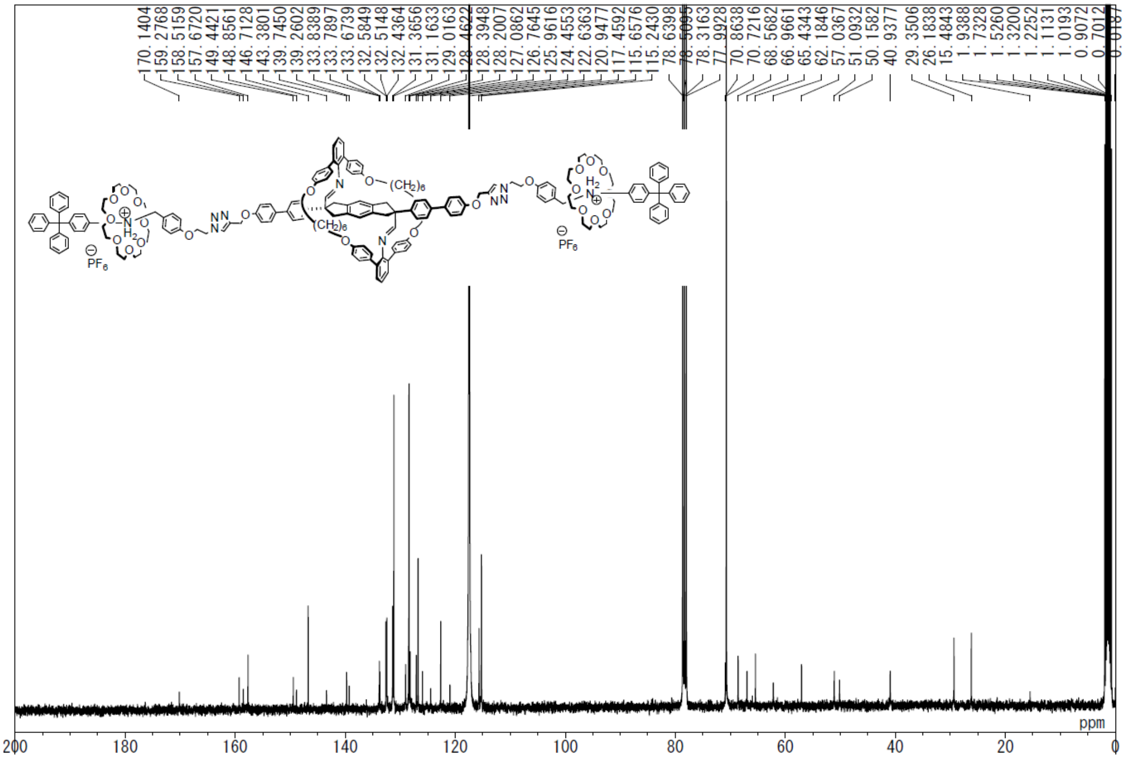

Figure S10. ^{13}C-NMR spectrum of **R1**.

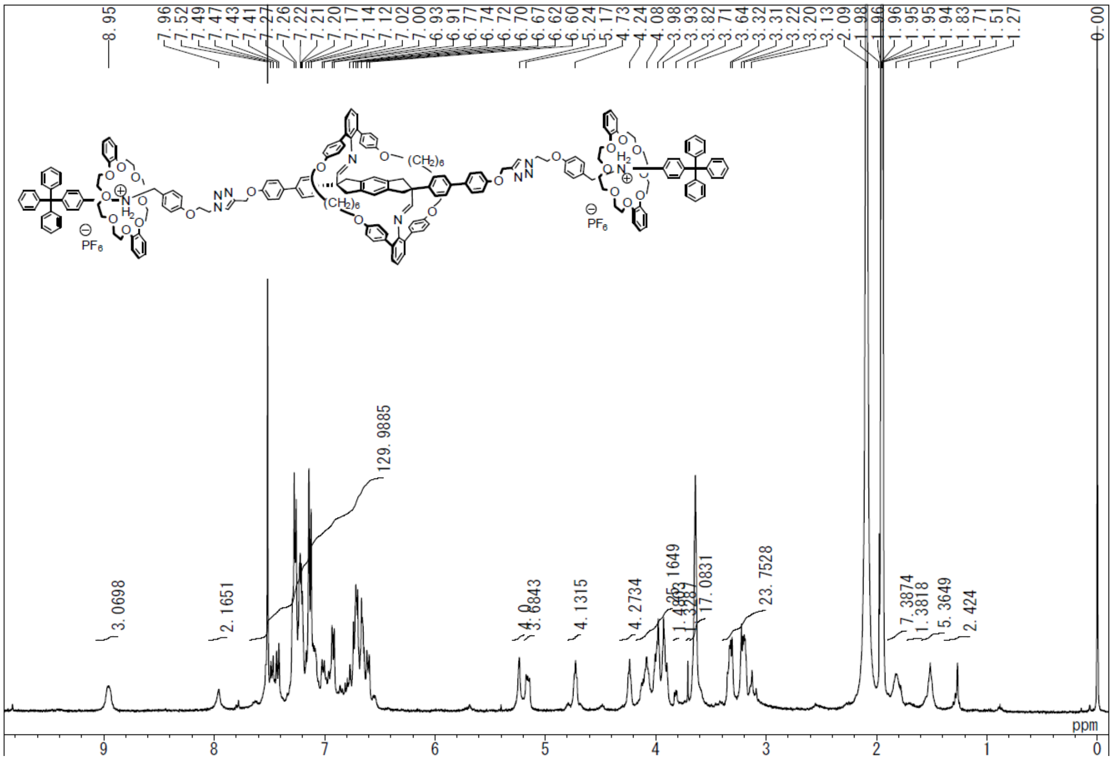

Figure S11. ^{1}H-NMR spectrum of **R2**.

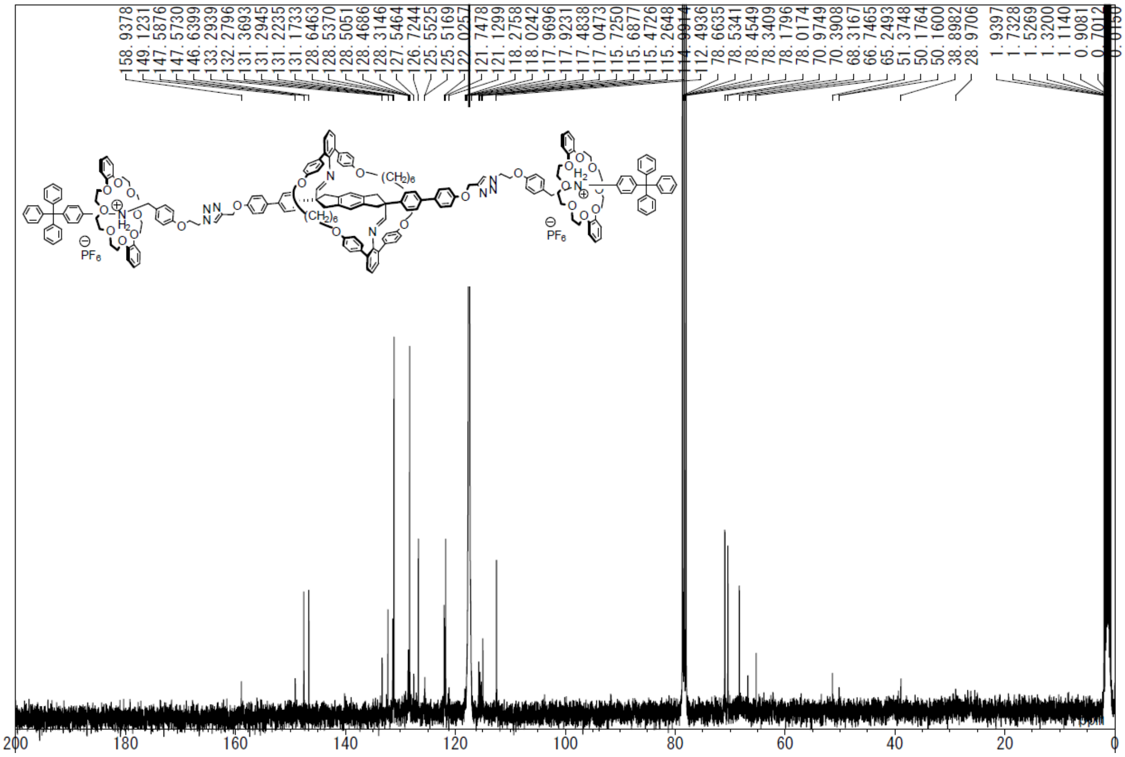

Figure S12. ^{13}C-NMR spectrum of **R2**.

2. Deslipping of Macrocycle from Imine-Bridged Heterorotaxane R2

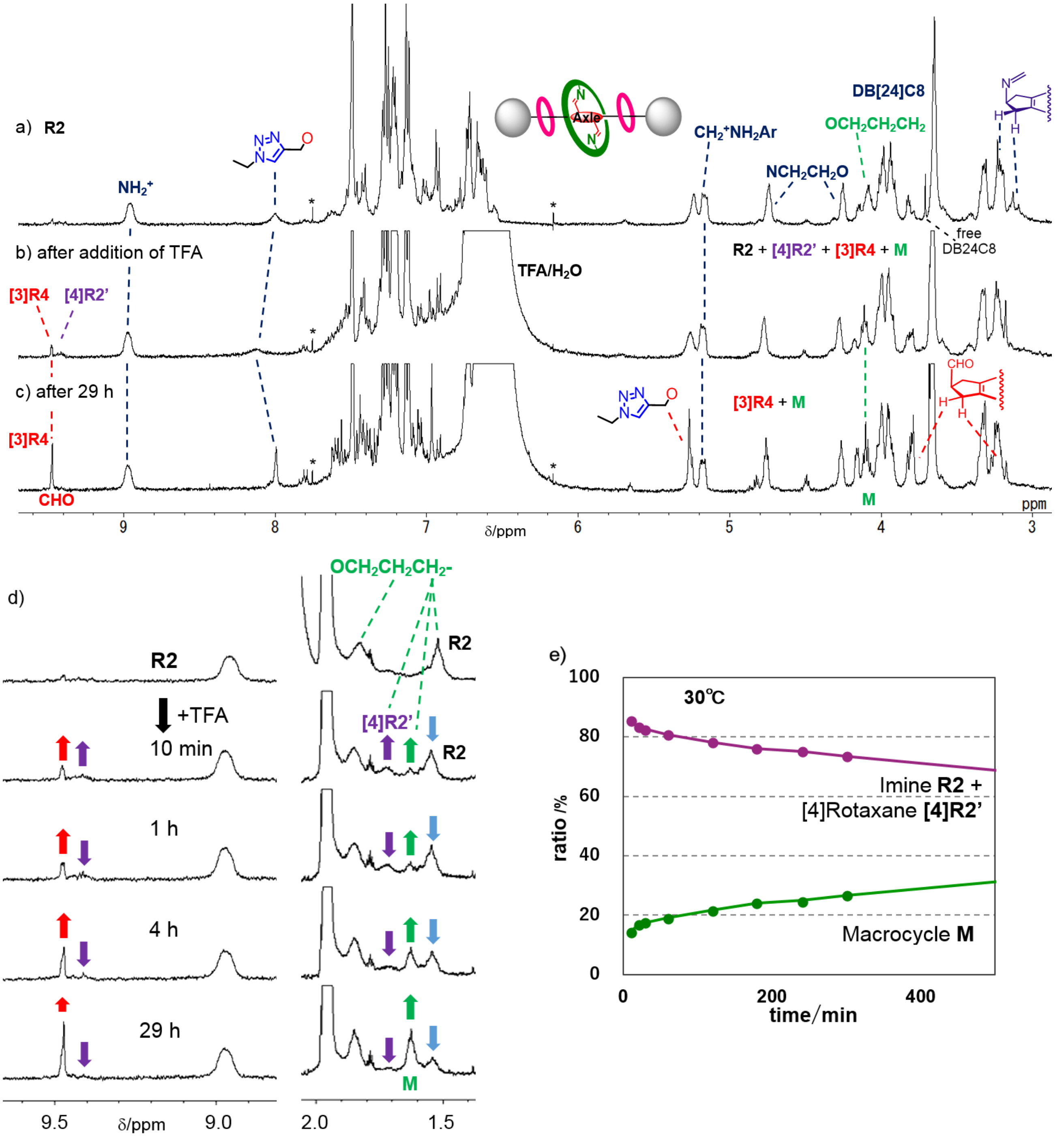

Figure S13. ^{1}H NMR spectra (400 MHz, $CDCl_3/CD_3CN$ at 303 K) of (a) imine-bridged heterorotaxane **R2**, (b) hetero[4]rotaxane **[4]R2'** and [3]rotaxane **[3]R4** generated upon the addition of TFA to a solution of **R2**, (c) [3]rotaxane **[3]R4** and macrocycle **M** after 29 h and (d) time-course of hetero[4]rotaxane **[4]R1'** and dethreaded macrocycle **M** from imine-bridged heterorotaxane **R2** upon imine hydrolysis. (e) Time-courses of ratios of imine-bridged heterorotaxane **R2** + hetero[4]rotaxane **[4]R2'** and dethreaded macrocycle **M** upon imine hydrolysis of **R2** at 30 °C.

3. Single crystal X-ray structural analysis of Macrocycle M

General. Suitable crystals for diffraction experiments of macrocycle **M** was obtained by recrystallization from a THF solution. The single crystal X-ray structure determination was performed on Rigaku XtaLAB mini (MoKα radiation, λ = 0.71075 Å). A numerical absorption correction (μ) was applied. The structure was solved by direct methods and refined by the full-matrix least-squares method on *F*2 with anisotropic temperature factors for non-hydrogen atoms. All the hydrogen atoms were located at the calculated positions and refined with riding. The disordered alkyl chain atoms of **M** were restricted by EADP. The disordered two THF molecules were removed by SQUEEZE program. Crystallographic data collection and refinement information is listed in Table S1.

Table S1. Crystal data and structure refinement parameters for **M**.

**Compound**	**M**
Data deposition	CCDC 2158687
Empirical formula	$C_{48}H_{50}N_2O_4$•2(C_4H_8O)
Formula weight	863.10
Temperature	173 K
Wavelength	0.71075 Å
Crystal system	monoclinic
Space group	*C*2/*c*
Unit cell dimensions a	25.771(14) Å
b	20.174(11) Å
c	18.521(10) Å
α	90 º
β	97.093(5) º
γ	90 º
Volume	9555(9) Å^3
Z	8
Density (calculated)	1.200 g/cm^3
Absorption coefficient Mu	0.532 mm^{-1}
F(000)	1200.0
Crystal size	0.40×0.25×0.15 mm^3
Theta range for data collection	2.216 º to 27.5 º
Index ranges	$-33<=h<=33$
	$-26<=k<=26$
	$-24<=l<=24$
Reflections collected	50531
Refinement method	Full-matrix least-squares on F^2
Data/restraints/parameters	10963/6/513
Goodness-of-fit on F^2	1.034
Final R indices	R_1 = 0.0876 [I>2σ(I)] , wR_2 = 0.2418 (all data)

4. Molecular Models of Macrocycle, Crown Ether and Endcap Moieties

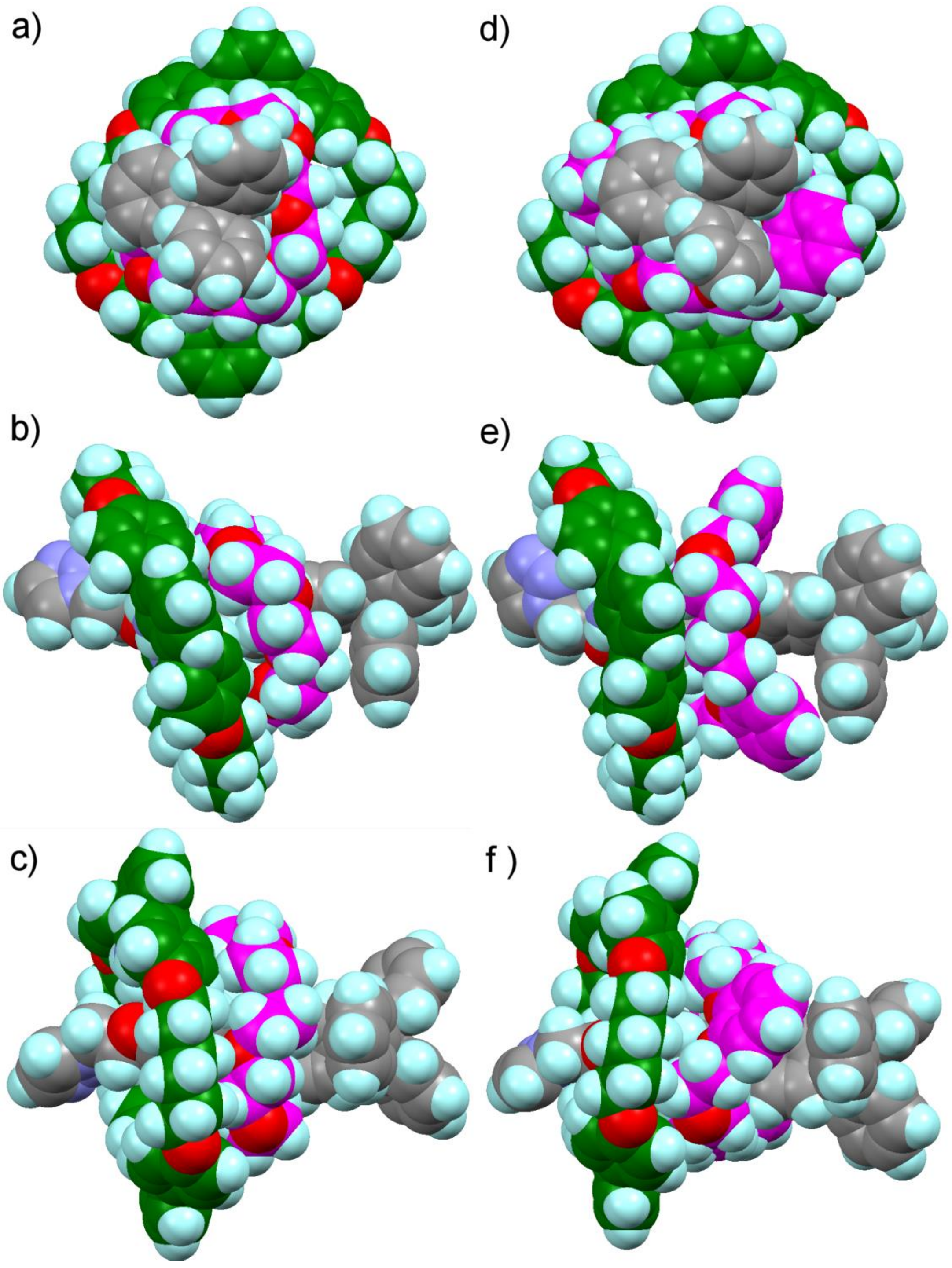

Figure S14. a) Front view, b) top view, and c) side view of the molecular model of macrocycle **M** and **24C8** on the axle moiety with an endcap. d) Front view, e) top view, and f) side view of the molecular model of macrocycle **M** and **DB24C8** on the axle moiety with an endcap. These molecular models (neither the most stable structure nor transition structure) were prepared by using the SPARTAN '14 to estimate the size of rings.

5. Kinetics and Thermodynamic Data for ‘Ring-over-Ring’ Deslipping

a)

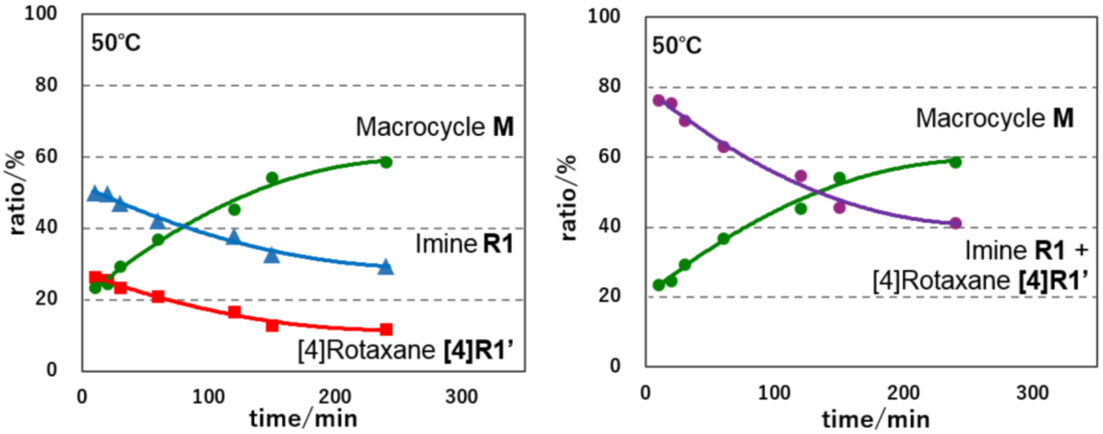

b)

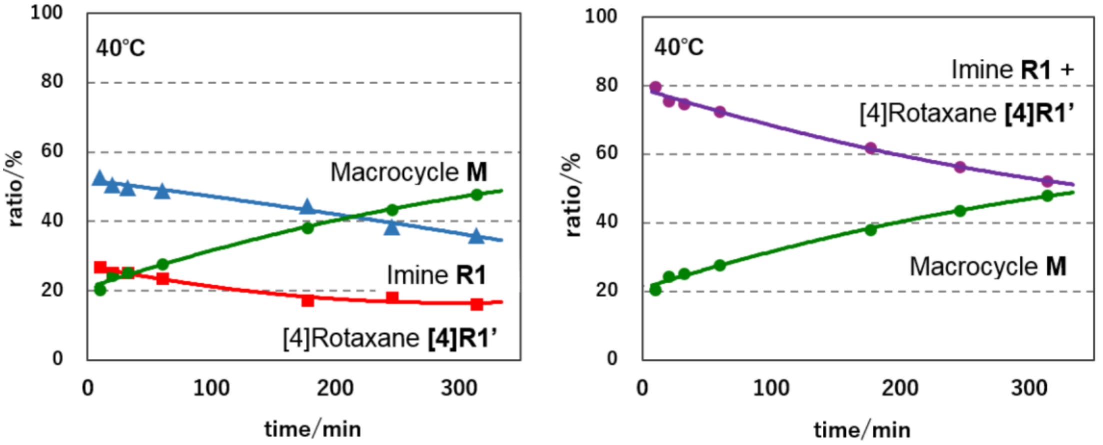

c)

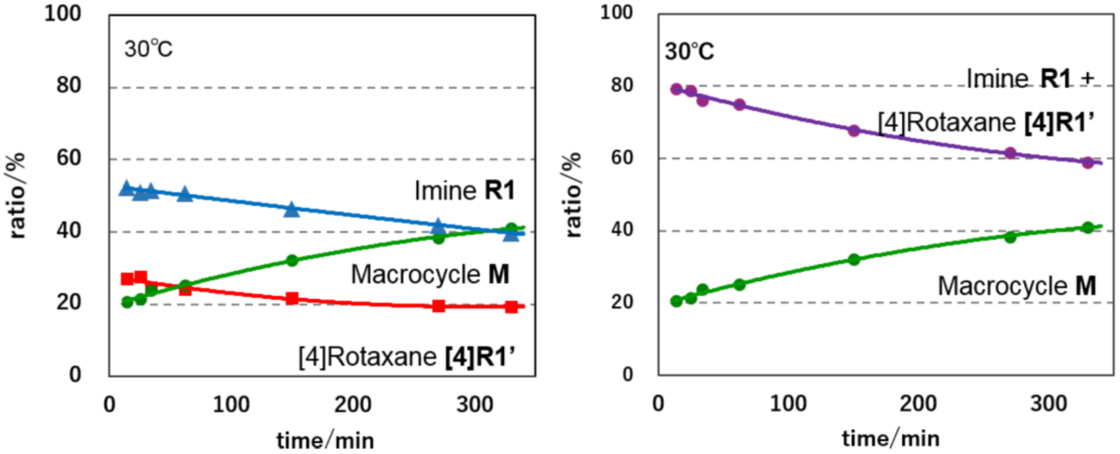

Figure S15. Time-courses of ratios of imine-bridged heterorotaxane **R1**, hetero[4]rotaxane **[4]R1’** and dethreaded macrocycle **M** upon imine hydrolysis of **R1** at a) 50, b) 40 and c) 30 °C. The curves are quadratic approximation for each plot.

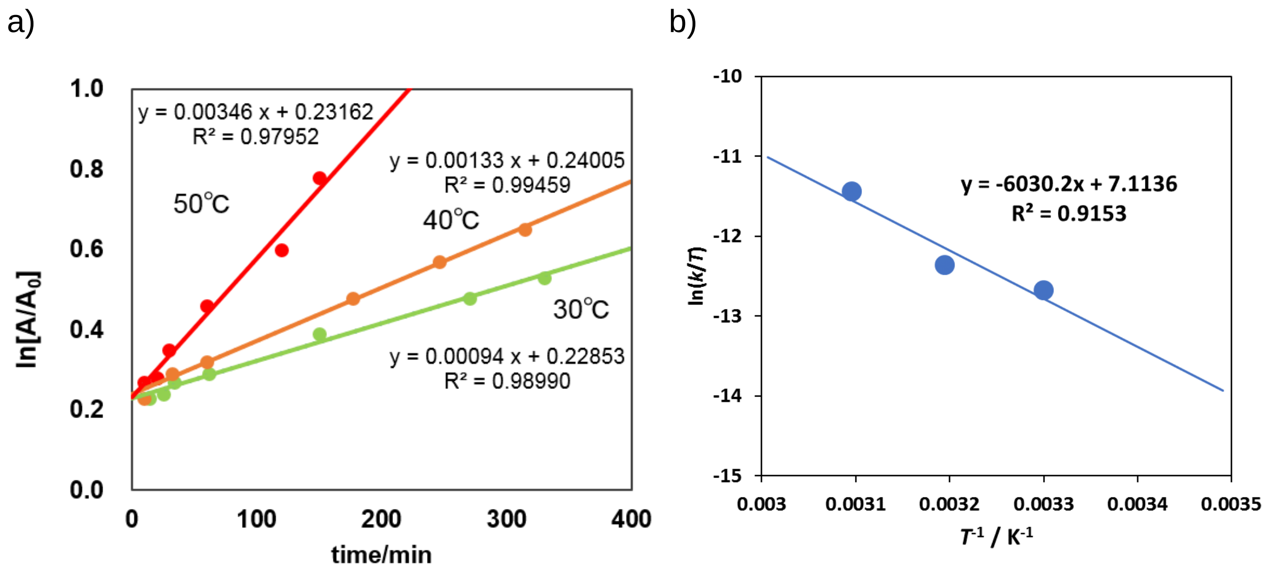

Figure S16. a) Pseudo-first-order plot of deslipped macrocycle **M** and b) Eyring-Polanyi plot of ‘ring-over-ring’ deslipping of macrocycle **M** from heterorotaxane **[4]R1’** upon imine hydrolysis.
